# Lead Clinical and Preclinical Antimalarial Drugs Can Significantly Reduce Sporozoite Transmission to Vertebrate Populations

**DOI:** 10.1128/AAC.03942-14

**Published:** 2014-12-23

**Authors:** L. M. Upton, P. M. Brock, T. S. Churcher, A. C. Ghani, P. W. Gething, M. J. Delves, K. A. Sala, D. Leroy, R. E. Sinden, A. M. Blagborough

**Affiliations:** a Department of Life Sciences, Imperial College London, South Kensington, London, United Kingdom; b Department of Infectious Disease Epidemiology, Imperial College London, St. Mary's Campus, London, United Kingdom; c Spatial Ecology and Epidemiology Group, Department of Zoology, University of Oxford, Oxford, United Kingdom; d Medicines for Malaria Venture, Geneva, Switzerland; e Jenner Institute, the University of Oxford, Oxford, United Kingdom

## Abstract

To achieve malarial elimination, we must employ interventions that reduce the exposure of human populations to infectious mosquitoes. To this end, numerous antimalarial drugs are under assessment in a variety of transmission-blocking assays which fail to measure the single crucial criteria of a successful intervention, namely impact on case incidence within a vertebrate population (reduction in reproductive number/effect size). Consequently, any reduction in new infections due to drug treatment (and how this may be influenced by differing transmission settings) is not currently examined, limiting the translation of any findings. We describe the use of a laboratory population model to assess how individual antimalarial drugs can impact the number of secondary Plasmodium berghei infections over a cycle of transmission. We examine the impact of multiple clinical and preclinical drugs on both insect and vertebrate populations at multiple transmission settings. Both primaquine (>6 mg/kg of body weight) and NITD609 (8.1 mg/kg) have significant impacts across multiple transmission settings, but artemether and lumefantrine (57 and 11.8 mg/kg), OZ439 (6.5 mg/kg), and primaquine (<1.25 mg/kg) demonstrated potent efficacy only at lower-transmission settings. While directly demonstrating the impact of antimalarial drug treatment on vertebrate populations, we additionally calculate effect size for each treatment, allowing for head-to-head comparison of the potential impact of individual drugs within epidemiologically relevant settings, supporting their usage within elimination campaigns.

## INTRODUCTION

Malaria is a devastating global human disease caused by protozoan parasites of the genus Plasmodium, which is exclusively transmitted by anopheline mosquitoes. Interventions including long-lasting insecticidal nets and improved access to care have substantially reduced global malaria morbidity and mortality over the last decade (1). In sub-Saharan Africa, approximately 40% of the population residing in areas with stable transmission is now estimated to experience an entomological inoculation rate (EIR) of less than one infectious bite per year (2). Despite these successes, the human burden remains high, with an estimated 219 million cases globally in 2013 (1). However, the tools currently available are insufficient to interrupt transmission in areas of high endemicity (3), and there remains growing concern over the spread of insecticide and parasite drug resistance (4). To achieve elimination, and ultimately eradication, there is therefore a need to develop novel tools that target the parasite at the weakest points in its life cycle. One option to achieve this is to target Plasmodium using either transmission-blocking drugs (TBDs) or transmission-blocking vaccines (TBVs) which could, either alone or in combination with other interventions, interrupt transmission or achieve local elimination of the parasite (5).

In recent years, a large number of potential new antimalarial TBDs have been discovered. Those that are comparatively advanced in the development pipeline include the synthetic endoperoxidase OZ439, which has proven activity against the asexual stages of the parasite (6) and is currently in phase IIa clinical trials, and the spiroindolone NITD609, which has previously demonstrated potent dose-responsive activity against the sexual stages of Plasmodium falciparum (7). These relatively new drugs add to the battery of more comprehensively analyzed TBDs, such as the clinically used antimalarial drug combination artemether-lumefantrine (A-L) and the 8-aminoquinoline primaquine. A-L is a widely used artemisinin combination therapy (ACT) approved for the treatment of uncomplicated P. falciparum malaria. Although it has been speculated previously that artemether is responsible primarily for the transmission-blocking activity of this ACT, lumefantrine has also been shown to inhibit mosquito transmission (7–9). Primaquine exhibits poor activity against asexual parasites but has established activity against mature gametocytes (8, 9) and is the only widely available drug capable of clearing mature P. falciparum gametocytes and hence preventing onward transmission (10, 11). The World Health Organization (WHO) has recently recommended the use of a single dose of 0.25 mg of primaquine/kg of body weight in addition to ACTs in malaria elimination programs (1). The use of primaquine in this manner is predicted to slow the emergence of artemisinin resistance while reducing toxicity concerns related to hemolysis in G6PD-deficient individuals. The effect of primaquine on reducing onward transmission remains unproven (12).

Given the current resurgent interest in interrupting malaria transmission (5, 13), a wide range of novel potential TBDs are currently undergoing assessment and subsequent triage in a number of transmission-blocking assays. Examples of these assays include multiple early/late stage gametocyte assays (14–20), exflagellation/male gamete assays (9, 21, 22), ookinete conversion assays (23–25), and sporozoite formation assays (26–28). Although typically cheap and of high to medium throughput, these assays have diverse and divergent outputs and usually yield data of low biological content. The current “gold standard” assay to assess transmission-blocking interventions, the standard membrane feeding assay (SMFA), is also heavily utilized in the current TBD development pipeline, typically assessing efficacy as a reduction in oocyst prevalence and intensity within the mosquito midgut. While all of these assays are undoubtedly valuable tools to identify and measure the potency of potential TBDs, none of them measure the desired endpoint of a TBD, namely, the reduction in the number of new malaria infections in the target vertebrate population (13). Instead, current methods measure convenient outputs, e.g., reduction in viable gametocytes (stage II to stage V) or reduction of parasite burden (oocyst or sporozoite stages) within the mosquito. It is unclear how reductions in these surrogate endpoints impact on subsequent mosquito-to-vertebrate transmission and, consequently, the number of new malaria infections or the reproductive number (*R_o_*). This strongly limits the potential translation of their impact, but assessments of impact on human populations are currently ethically challenging. Furthermore, future regulatory approval for TBDs is likely to require provision of data directly demonstrating their impact in vertebrate populations. To address these issues, we developed a reliable and cost-effective transmission-based mouse-to-mouse population model to assess how individual (or combinations of) TBDs impact the number of secondary cases of malaria over cycles of transmission within a controlled laboratory setting (28). The model simulates a range of different transmission intensities by varying the number of mosquito bites on each naive mouse (referred to as the mosquito biting rate [MBR]). The use of multiple MBRs allows us to estimate the effect size of the intervention, which is a measure of the ability of the treatment to reduce *R_o_* (29). By calculating this important parameter for individual TBDs, we aim to link “traditional” lab-derived transmission-blocking assays to the potential practical impact at the population level.

Here, we report the evaluation of a number of clinical and preclinical TBDs in this population model over an entire transmission cycle. We demonstrate the impact of individual TBDs on both mosquitoes and subsequent mice and estimate the effect sizes for each drug regime, allowing, for the first time (to our knowledge), head-to-head comparison of the potential impact of individual TBDs on subsequent malarial infections and disease incidence within vertebrate populations.

## MATERIALS AND METHODS

### Drug treatments.

Artemether-lumefantrine (A-L; Coartem), OZ439, and NITD609 were tested in duplicate at a concentration equivalent to 3 times the 90% effective dose (ED_90_; reduction of asexual parasitemia by 90% *in vivo*). Primaquine was tested at 4 different concentrations (12, 6, 1.25, and 0.25 mg/kg). The triple combination of A-L and primaquine was tested using 2 different primaquine concentrations (12 and 0.25 mg/kg). All treatments consisted of a single dose of drug. Each experiment included three control treatments. The diluting agent for all test drugs, 1% methyl cellulose (Sigma-Aldrich no. M7140), was used as a negative control (referred to as “no-drug control”). Sulfadiazine (Sigma-Aldrich no. S6387) and atovaquone (ATV; Sigma-Aldrich no. A7968) were used as negative and positive drug controls, respectively (28, 30). The inability of sulfadiazine to reduce gametocytemia under the conditions tested is demonstrated in Fig. S2 in the supplemental material. All treatments were delivered by oral gavage except sulfadiazine and ATV, which were administered by intraperitoneal (i.p.) injection. Table 1 summarizes the treatments and doses used.

**TABLE 1:** Drug treatments used in the mouse-to-mouse model^*a*^

Drug(s)	Dose(s)	Diluting agent	Volume(s) (μl)	Delivery method
No drug (control)	1% methyl cellulose	Water	100–200	Oral
SD (control)	8.4 mg/kg	Water	100	i.p.
ATV (control)	0.3 mg/kg	DMSO	100	i.p.
A-L	57 and 11.8 mg/kg for A and L, respectively	1% MC	50 each	Oral
PQ	12, 6, 1.25, or 0.25 mg/kg	1% MC	100	Oral
A-L and PQ	57 and 11.8 mg/kg for A and L, respectively, and 12 or 0.25 mg/kg PQ	1% MC	50 for A and L and 100 for PQ	Oral
OZ439	6.5 mg/kg	1% MC	100	Oral
NITD609	8.1 mg/kg	1% MC	100	Oral

a Drugs were prepared in either water, dimethyl sulfoxide (DMSO), or 1% methyl cellulose (MC), as stated. Either 100 or 200 μl of 1% MC was used for the no-drug control, depending on the maximum drug volume for that experiment. Treatments were delivered by oral gavage or i.p. injection as indicated, 24 h prior to mosquito feeding. ATV, atovaquone; PQ, primaquine; A-L, artemether and lumefantrine; SD, sulfadiazine.

### Mouse-to-mouse transmission model.

**FIG 1:**
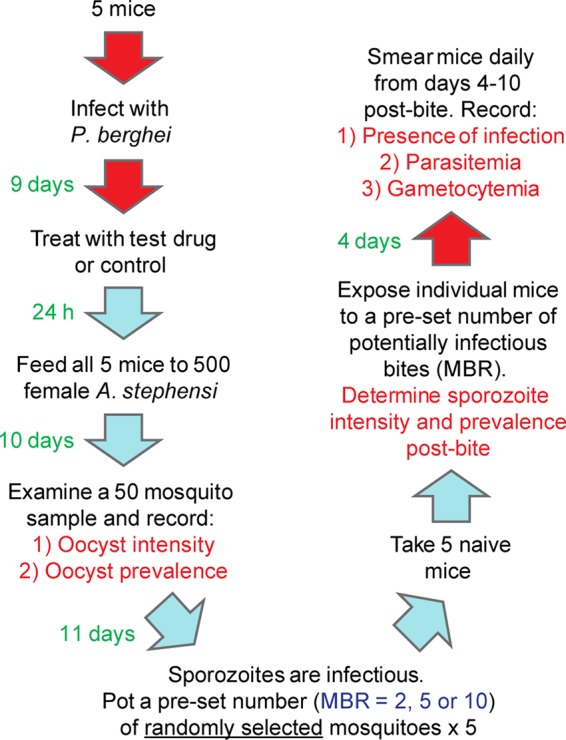
The mouse-to-mouse model. A schematic illustrating the experimental design of the mouse-to-mouse model using P. berghei and A. stephensi. For each treatment group, the drug was administered to five P. berghei-infected mice which were used to feed a cage of 500 female A. stephensi mosquitoes 24 h later. After 10 days, oocyst intensity and prevalence were determined. When sporozoites were maximally infectious (21 days after feeding [28]), individual naive mice were bitten by either 2, 5, or 10 mosquitoes (the mosquito biting rate [MBR]). Salivary glands were dissected postbite to determine sporozoite intensity and prevalence. The presence of infection in the peripheral blood of challenged naive mice (i.e., the number of secondary malarial infections) was monitored daily for 10 days postbite, with parasitemia, gametocytemia, and time to patency recorded.

For each treatment regime, mouse-to-mouse transmission was performed in duplicate (except primaquine, where individual doses were tested in single experiments) to maximize statistical significance in balance with cost, with general parasite maintenance carried out as previously described (28). A schematic of the model is shown in Fig. 1. Briefly, for each treatment group, five female TO mice (6 to 8 weeks old) were infected with Plasmodium berghei clone 507cl1 (31) by syringe inoculation (i.p.). All care and handling of animals was in accordance with the Guidelines for Animal Care and Use prepared by Imperial College London. At day 9 postinfection, tail blood drops were examined for the presence of exflagellation, and mice were given the appropriate drug/control treatment by the appropriate route (see Table 1). Blood stage infections were monitored on Giemsa-stained tail blood smears, before and 24 h after treatment, as previously described (28).

Ten days postinfection and 24 h posttreatment, the mice were anesthetized and exposed to 500 starved female Anopheles stephensi (line sd 500) mosquitoes. Mosquitoes that did not take a blood meal were discarded, and the remaining mosquitoes were maintained on 8% (wt/vol) fructose and 0.05% (wt/vol) *p*-aminobenzoic acid at 19°C and 80% relative humidity. On day 10 postfeeding, midguts were dissected from a random sample of 50 mosquitoes per cage and oocyst prevalence (percentage of infected mosquitoes) and intensity (mean number of parasites per midgut) were recorded. For each treatment group, oocyst prevalence and mean oocyst intensity were compared to the no-drug control group to calculate “classical” inhibition of transmission. All mosquito dissections to assess oocyst intensity and prevalence were performed under randomized and double-blind conditions.

The remaining mosquitoes were maintained until 21 days postinfection, when salivary gland sporozoites were at their peak of infectiousness (28). Individual anesthetized naive mice were exposed for 20 min to predetermined numbers of potentially infectious mosquitoes, randomly selected from the appropriate mosquito population. For each treatment group, five individual naive mice were exposed to either 2, 5, or 10 mosquito bites. This range of MBRs (simulating different transmission intensities) is necessary to calculate effect size (see below). Successful feeding was confirmed by the presence of blood in the mosquito abdomen. Where necessary, additional mosquitoes were given the opportunity to feed until the required number of successful bites was achieved. Postfeeding, all mosquitoes were individually dissected to determine the prevalence of salivary gland sporozoites. Glands were directly dissected onto a glass slide and covered with a coverslip, and intensities were scored on a log scale (score of 0 = no sporozoites visible; 1 = 1 to 10 sporozoites visible; 2 = 10 to 100 sporozoites visible; 3 = 100 to 1,000 sporozoites visible; 4 = 1,000+ sporozoites visible). For each treatment group, sporozoite prevalence and mean sporozoite intensity were compared to the no-drug control group to calculate inhibition. The “bitten” mice were allowed to recover and maintained for 10 days postfeeding. Daily tail blood smears were performed from days 4 to 10 to establish prepatency, parasitemia, and gametocytemia.

Data reporting proportion of biting mosquitoes with salivary gland sporozoites, oocyst intensity, and oocyst prevalence from all experiments are included in Dataset S1 in the supplemental material.

### Statistical analysis.

Generalized linear mixed models were used to estimate the overall effectiveness of the different interventions combining data from all repeat replicates (32). Efficacy of the treatment (compared to the no-drug control) was included as a fixed effect, while the mouse-to-mosquito transmission parameters were included as random effects. For the oocyst and sporozoite prevalence data, we assumed a binomial error structure, while for oocyst, asexual parasite, and gametocyte intensity, we used a zero-inflated negative binomial distribution. The impact on sporozoite intensity was assessed by looking at differences in sporozoite score, which was assumed to follow a Poisson distribution. Ninety-five percent confidence intervals were estimated by bootstrapping, and the model was selected using a likelihood ratio test.

The overall effectiveness of an intervention over one round of transmission (from mouse to mosquito to mouse) can be quantified by estimating its ability to reduce the basic reproduction number. This has been termed the effect size (29). If it is assumed that that all infectious mosquitoes are equally infectious, this can be estimated by fitting a chain binomial model (33). A full description of the methodology is given in reference 28. The models were fitted to the data using maximum likelihood methods and the 95% confidence interval estimates obtained from the likelihood profile.

### Ethical statement.

All procedures were performed in accordance with the terms of the United Kingdom Animals (Scientific Procedures) Act (PPL 70/7185) and were approved by the Imperial College Ethical Review Committee. The Office of Laboratory Animal Welfare (OLAW) Assurance for Imperial College covers all Public Health Service (PHS)-supported activities involving live vertebrates in the United States (A5634-01).

## RESULTS

### Mouse-to-mouse model and drug treatments.

Antimalarials and clinical TBD candidates (Table 1) were screened for malaria transmission-blocking efficacy using the mouse-to-mouse model (Fig. 1). Further details regarding treatment and controls are described in Materials and Methods. All raw data generated from individual experiments are given in Dataset S1 in the supplemental material.

### Effect on parasite transmission to, and within, the mosquito vector.

**FIG 2:**
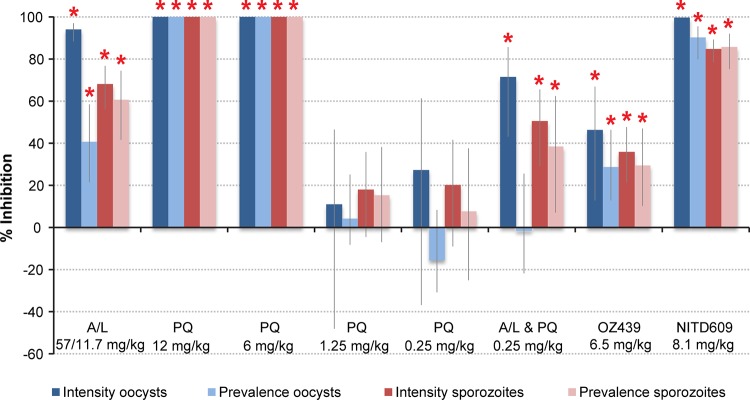
Transmission-blocking effect of drug treatments within the mosquito vector. Inhibition of oocyst intensity and prevalence (dark- and light-blue bars, respectively) and sporozoite intensity and prevalence (dark- and light-red bars, respectively) in the mosquito vector after feeding on drug-treated infected mice. Vertical lines denote 95% confidence interval estimates. Inhibition was calculated relative to the no-drug control. Red asterisks indicate statistically significant (*P* < 0.05) levels of inhibition.

The impact of drugs administered to groups of P. berghei-infected mice on the development of oocysts in the midgut and sporozoites in the salivary glands of Anopheles stephensi mosquitoes was recorded. The percentages of inhibition of oocyst and sporozoite intensity and prevalence for each treatment regime compared to those of the no-drug control were calculated (Fig. 2). For the no-drug control, overall oocyst intensity was 47.7 (standard error of the mean [SEM] = 4.0) and oocyst prevalence was 77.7%.

Compared to the no-drug control, A-L at 57 mg/kg-11.8 mg/kg significantly reduced parasite intensity and prevalence in the mosquito. Oocyst intensity was inhibited by 94% (95% confidence interval [CI] of 88 to 97), oocyst prevalence by 41% (95% CI of 22 to 58), and ensuing sporozoite intensity and prevalence by 68% (95% CI of 56 to 77) and 61% (95% CI of 42 to 74), respectively. Overall oocyst intensity for A-L was 6.78 (SEM = 3.2), and oocyst prevalence was 69.5%.The impact of primaquine was clearly dose dependent. At 12 mg/kg (independently or in combination with A-L) and 6 mg/kg, oocyst and sporozoite development was completely blocked, whereas the drug had no significant effect at 1.25 and 0.25 mg/kg (1.25 mg/kg, oocyst intensity = 39.1 [SEM = 6.1] and oocyst prevalence = 80.6%; 0.25 mg/kg, oocyst intensity = 42.9 [SEM = 6.1] and oocyst prevalence = 79.1%). In this model, the triple combination, containing primaquine (0.25 mg/kg), was marginally but significantly (*P* = 0.0014) less efficacious than A-L alone (inhibition in oocyst intensity of 71% [95% CI of 43 to 86] compared to 94% [95% CI of 88 to 97], mean oocyst intensity = 16.6 [SEM = 3.2], oocyst prevalence = 69.5%). OZ439 significantly inhibited both oocyst and sporozoite intensity and prevalence, though the efficacy was relatively modest (46% against oocyst intensity [95% CI of 13 to 67], 29% against oocyst prevalence [95% CI of 13 to 46], mean oocyst intensity = 26.6 [SEM = 4.1], oocyst prevalence = 56.3%). NITD609 significantly inhibited both oocyst and sporozoite intensity and prevalence by ≥85% (mean oocyst intensity = 0.1 [SEM = 0.03], oocyst prevalence = 8.3%). The atovaquone (ATV) positive control completely blocked the mosquito stages of infection, whereas the sulfadiazine negative control, compared to the no-drug control, had no detectable effect on vertebrate-mosquito transmission.

### Effect on transmission to secondary vertebrate populations at different mosquito biting rates.

The impact of individual drug regimens on malarial transmission from infected mosquitoes to a secondary population of naive mice at different transmission intensities (MBRs of 2, 5, and 10 bites) was assessed (the use of differing MBRs permits the estimation of the key output, namely, effect size). Table 2 presents the impact of each compound on mouse infection, compared to that of the drug-free controls, illustrating the prepatent period between sporozoite inoculation and observation of asexual parasites and the percentage reduction in parasitemia and gametocytemia at day 10 postbite. The number of secondary infections (infection prevalence of blood stage infection) was assessed as the prevalence of blood stage infection in mice 10 days after mosquito bites.

**TABLE 2:** Effect of drug treatments on transmission to secondary mouse populations^*a*^

Drug(s) (dose)	Prepatent period in days (±SEM)	% inhibition		
		Infection prevalence	Parasitemia day 10	Gametocytemia day 10
No drug	5.7 (±0.31)	NA	NA	NA
A-L (57 and 11.8 mg/kg)	6.9 (±0.65)	57.9^*b*^	50.4^*b*^	25.8
PQ (12 mg/kg)	Not infected	100.0^*b*^	100.0^*b*^	100.0^*b*^
PQ (6 mg/kg)	Not infected	100.0^*b*^	100.0^*b*^	100.0^*b*^
PQ (1.25 mg/kg)	5.4 (±0.13)	0.0	4.4	37.4
PQ (0.25 mg/kg)	5.9 (±0.19)	−9.1	34.2^*b*^	7.7
A-L and PQ (12 mg/kg)	Not infected	100.0^*b*^	100.0^*b*^	100.0^*b*^
A-L and PQ (0.25 mg/kg)	6.3 (±0.53)	27.3	34.5	54.0
OZ439 (6.5 mg/kg)	6.1 (±0.45)	12.0	13.1	−12.4
NITD609 (8.1 mg/kg)	Not infected	100.0^*b*^	100.0^*b*^	100.0^*b*^

a The prepatent period reduction in infection prevalence and reduction in asexual and sexual infection intensity (in infected mice) are illustrated. Percentage of inhibition was calculated relative to the no-drug control. NA, not applicable. Results from all biting rates are included.

b Statistical significance (calculated using Fisher's exact test for percent inhibition in infection prevalence and using bootstrapping, 10,000 replicates, for percent inhibition in parasitemia and gametocytemia at day 10).

Artemether-lumefantrine significantly reduced the number of secondary infections (58% reduction) compared to that of the no-drug control (Table 2). Mice that did become infected exhibited a longer prepatent period (6.9 ± 0.65 versus 5.7 ± 0.31 days) and a significantly lower parasitemia at day 10 (50%, 95% CI of 39 to 60) than mice infected in the control arm. Primaquine given at 12 and 6 mg/kg completely blocked transmission to a secondary mouse population but did not exhibit a potent effect at doses of 1.25 and 0.25 mg/kg. Primaquine given at a dose of 0.25 mg/kg significantly inhibited parasitemia in infected mice by 34% (95% CI of 11 to 51%) despite the lack of a detectable impact when observing oocyst intensity/prevalence in the mosquito population. Conversely, the triple combination of A-L and primaquine at doses of 12 or 0.25 mg/kg resulted in complete transmission blockade or no impact, respectively, consistent with the measured impact on oocyst and sporozoite numbers. Under the transmission settings examined and at the dose administered, OZ439 had no significant impact on the number of secondary infections, parasitemia or gametocytemia, despite the significant inhibition observed in both oocyst and sporozoite intensity and prevalence. NITD609 resulted in 100% transmission blockade to secondary populations, despite a low number of oocysts and sporozoites being observed in the mosquito population after treatment (Fig. 2). As previously noted (28), increasing the MBR, not unexpectedly, increased the proportion of mice infected (see Dataset S1 in the supplemental material).

### Effect size of TBD treatment.

The estimated effect size for each drug is shown in Table 3. The drugs can be broadly divided into three categories: those with total (100%), moderate (25% to 60%), and low (<10%) estimated effect sizes. As with the ATV positive control, we estimated a 100% effect size for NITD609 (despite a low number of oocysts and sporozoites being observed in mosquito populations [Fig. 2]) and for primaquine at 12 and 6 mg/kg, indicating that at the maximum exposure considered (MBR = 10), these drugs would result in elimination from these laboratory populations. Drugs with a moderate estimated effect size included A-L, OZ439, and primaquine at 1.25 mg/kg (58%, 57%, and 29%, respectively, at the stated doses), while the estimated effect size for primaquine (alone) at 0.25 mg/kg was comparatively low (8%).

**TABLE 3:** Effect size of individual drug treatments^*a*^

Effect size	Drug(s) (dose)	Effect size (95% CI)
Total	ATV (0.3 mg/kg)	100 (96–100)
	PQ (6 mg/kg)	100 (96–100)
	NITD609 (8.1 mg/kg)	100 (96–100)
	PQ (12 mg/kg)	100 (58–100)
	A-L and PQ (12 mg/kg)	100 (58–100)
Moderate	A-L (57 and 11.8 mg/kg)	58 (19–86)
	OZ439 (6.5 mg/kg)	57 (31–75)
	A-L and PQ (0.25 mg/kg)	42 (20–99)
	PQ (1.25 mg/kg)	29 (−29–69)
Low	PQ (0.25 mg/kg)	8 (−18–48)
	SD (8.4 mg/kg)	−0.1 (−0.8–0.3)

a Drug treatments have been ranked in order of effect size and transmission-blocking efficacy. Treatments have been broadly divided into three groups: drugs with total (100%) effect size, drugs with a moderate effect size, and drugs with a low effect size. ATV, atovaquone; PQ, primaquine; A-L, artemether-lumefantrine; SD, sulfadiazine.

## DISCUSSION

Using a mouse-to-mosquito-to-mouse population transmission model, we have analyzed five clinical and preclinical antimalarial drug candidates for transmission-blocking activity, individually assessing impact upon both mosquito (oocyst and sporozoite) and vertebrate (asexual and sexual blood stage) phases of development. This allows for the head-to-head comparison of efficacy and the estimation of effect size, thereby measuring the potential ability of TBDs to reduce *R_o_*. In this manner, we can simply triage individual drugs by their potential impact on vertebrate populations. The TBDs examined can be broadly divided into three categories: those with total (100%), moderate (25% to 60%), or low (<10%) estimated effect sizes.

A 100% effect size was estimated when examining the ability of the novel antimalarial drug candidate NITD609 to block malarial transmission. As an inhibitor of Plasmodium protein synthesis, NITD609 is known to act against multiple parasite life stages (7, 34) and has previously demonstrated excellent potency against P. falciparum and P. berghei. In previous studies, examining the asexual form of the parasite, a single oral dose of NITD609 at 100 mg/kg was reported to completely cure P. berghei-infected mice, whereas 30 mg/kg cured 50% of mice and 10 mg/kg could not clear infection (34). In terms of activity as a TBD, it has previously been shown to inhibit both early and late gametocyte development in a dose-dependent manner between 5 and 500 nM (7). NITD609 demonstrates efficacy in an *in vitro* P. berghei ookinete conversion assay, with a 50% inhibitory concentration (IC_50_) of 3 μM, and strongly inhibits transmission to mosquitoes in the SMFA (7). Here, NITD609 showed excellent potential as a TBD. At a dose of 8.1 mg/kg, NITD609 exhibited a potent (but not total) ability to inhibit transmission from mouse to mosquito and completely blocked progression of the life cycle to subsequent mice with a single dose. As NITD609 belongs to a novel, synthetic class of antimalarials, the spiroindolones, if approved, it has strong prospects for future use when targeting artemisinin-resistant parasites. It is the first antimalarial with a novel mode of action to enter phase IIa trials in the last 20 years. Our results, combined with impressive data reported previously, clearly demonstrate that NITD609 has exciting potential to be used as a potent single-dose TBD in the near future. A 100% effect size was also observed when examining the transmission-blocking efficacy of the control drug ATV, supporting previous studies showing potency (28). ATV is commonly administered with proguanil (Malarone) for treatment and prevention of malaria; however, resistance against the cytochrome *bc*_1_ complex that ATV targets is considered to be widespread and comparatively easily generated (35).

Primaquine, a widely utilized antimalarial, additionally generated a 100% estimated effect size at higher dosages (12 and 6 mg/kg). Its potent impact is strongly dose dependent; any induced transmission-blocking effect in both mosquito (Fig. 2) and mouse (Table 2) populations is clearly reduced at the lower concentrations examined (1.25 and 0.25 mg/kg), with moderate or low effect sizes generated correspondingly (29% or 8%, respectively). In previous studies, primaquine has been reported to reduce gametocyte carriage in combination with an ACT, with a wide range of studies reporting a significant reduction compared to that of the ACT alone (10, 36–41). The infectiousness of treated gametocytes to mosquitoes, or onward transmission and impact on new vertebrate infections, was not examined within any of these studies. Two older studies have directly examined the ability of primaquine to directly inhibit transmission in a small number of patients (*n* = 2) (42, 43). In these studies, a single dose of 45 mg of primaquine base proved to inhibit onward transmission from human to human (1 from a group of 4 individuals developed patent P. falciparum infection posttreatment at a single MBR of 75). Despite this raft of largely positive findings, the transmission-blocking efficacy of primaquine, particularly at lower doses and in combination with an ACT, remains poorly understood (1, 12, 44, 45). Our results corroborate previously observed dose dependency, although the doses at which we identified a significant effect were considerably higher. We observe no additional benefit of adding primaquine (at 0.25 mg/kg) to the A-L combination, consistent with two recent Cochrane reviews which concluded that there was no clear evidence as to whether primaquine (in combination with an ACT) can directly reduce onward transmission in an area where malaria is endemic, even if it significantly reduces gametocyte prevalence (44, 45). In light of these, and our, findings, the role of primaquine as a TBD in malaria treatment should continue to be examined carefully in subsequent studies, especially at low dosages. Several valuable clinical trials to determine the most effective dosing are under way (10).

A moderate effect size was observed when examining two further antimalarials, A-L and OZ439. A-L is a common ACT currently approved for treatment of uncomplicated P. falciparum malaria. Our data demonstrated only a moderate transmission-blocking effect of A-L at the tested dosages on P. berghei transmission to mosquitoes, which translated into a significant reduction in secondary malaria infections in mice. This is consistent with previous studies of P. falciparum in humans given this ACT as first-line therapy, which have demonstrated reduction in both gametocyte carriage posttreatment and onward transmission to mosquitoes following A-L treatment (11, 36, 46–50). The data described here further demonstrate the potential impact of A-L to achieve a substantial reduction in malaria infection within vertebrate populations at the dosages tested. Previous studies have discussed the translation of laboratory-based transmission of Plasmodium compared to transmission in the field (28, 51, 52). Translation of an effect size into impact in epidemiological settings requires further consideration of the mode of delivery, coverage, and the balance between reducing onward transmission and protection from reinfection (53). Nevertheless, an estimated effect size of 58% suggests that use of such drugs could lead to significant reductions in transmission if deployed appropriately in low-transmission settings.

Our results confirm that the endoperoxide, OZ439, has potential as a transmission-reducing drug. OZ439 has several advantages over artemisinin derivatives, including prolonged plasma exposure, higher potency, and its stable, synthetic nature (6). It has shown comparable efficacy in the treatment of P. falciparum and Plasmodium vivax patients (6), is undergoing phase IIa clinical trials, and is intended for use as a single-dose combination therapy for acute malaria. It has previously been demonstrated to completely cure P. berghei-infected mice (blood stage infection) with a single oral dose of 20 mg/kg (6) but has no effect on P. berghei ookinete development in *in vitro* assays at 10 μM, suggesting it functions prior to the ookinete stage (20). It has additionally demonstrated a potent effect in the SMFA with P. falciparum at 10 μM (20). Here, when administered 24 h prefeed at the tested dose of 6.5 mg/kg, OZ439 significantly reduced oocyst and sporozoite development in the mosquito but did not significantly reduce the number of secondary mouse infections. Despite this, when mosquito and mouse data were collated and examined using a chain-binomial model, a moderate effect size was estimated, comparable to that observed when using A-L at 3× ED_90_. Recent studies using SCID mice have suggested promising results against asexual blood stage infection using a dose of 19.5 mg/kg. Future studies should aim to complement these results by assessing the transmission-blocking efficacy of OZ439 at this higher dose. Additionally, evaluation of potential partner drugs for OZ439 is under way, with the aim of increasing the transmission-blocking potency of treatment and thus combating potential emerging resistance to the compound in the future.

The use of the mouse-to-mouse model addresses key gaps in our existing knowledge (54) that other transmission-blocking assays cannot, being the only fully *in vivo* model that measures the impact of an intervention on mosquito-to-vertebrate and vertebrate-to-vertebrate transmission. The resulting generation of effect size can be subsequently used within mathematical models of malaria transmission to predict the public health significance of individual TBDs in different settings and in combination with different interventions. These studies will, as data accumulate, also allow us to correlate “standard” transmission-blocking assay outputs (e.g., reduction in oocyst intensity/prevalence) to reduction in parasite prevalence/intensity in subsequent vertebrate populations. The use of the tractable rodent malaria parasite P. berghei provides a safe, cost-effective, and robust population model to examine these parameters. We are fully aware that any findings using rodent parasites require validation with respect to human malaria parasites; however, early-stage efficacy trials of this type using human volunteers are currently technically and ethically impossible (13). We are additionally aware that differences in drug pharmacokinetics (PK) between mice and humans are critical to any field extrapolation of the data (55, 56). Recognizing that the rate of drug clearance in rodents is approximately three times faster than that in humans (55–57), drugs given to rodents at 3× ED_90_, in comparison with an equivalent allometric dose in humans, will potentially result in lower levels of active circulating TBD 24 h posttreatment, potentially underpredicting the impact of compounds. To enhance our understanding of the biological process of metabolism and clearance of specific drugs, it would be advantageous for future studies to compare PK data for individual TBDs in rodents and humans at multiple time points posttreatment.

The systematic development, examination, approval, and widespread use of new antimalarial TBDs will require phase 3 trials, with successful outcome determined as the reduction in transmission measured by decreased incidence of malarial infection. Given the exceptionally high costs of such studies, and the subsequent ethical considerations, it is both important and logical to set realistic go/no-go criteria for efficacy before such trials. It is additionally highly advantageous to triage potentially effective drugs by head-to-head comparison prior to development to this level. The studies described here assist this comparison in a cost-effective, ethical, safe, and robust manner. Our results demonstrate that different TBDs have various transmission-blocking potencies at both the mosquito, vertebrate, and population levels, and hence successful formulation for their field utilization will differ in various transmission settings. The intelligent use of specific antimalarial drugs will require careful consideration of TBD efficacy, effect size, and transmission intensity/EIR in targeted areas. Even drugs with high transmission-blocking levels are likely to have the greatest impact if delivered as part of focal case detection strategies in low-transmission settings in which a high proportion of the infectious reservoir is treated (58, 59). We directly compare the ability of currently utilized (A-L and PQ) and novel preclinical (OZ439 and NITD609) antimalarials to reduce parasite intensity and prevalence at multiple transmission settings. We examine these crucial outputs not only within the mosquito but also following transmission to subsequent vertebrate populations, demonstrating their ability to act as potent, single-dose TBDs. Our data suggest that, of the compounds and combinations tested, PQ (at >6 mg/kg) alone, or in combination with A-L, and NITD609 (8.1 mg/kg) may have comparatively wider utility than other TBIs in reducing malaria transmission within populations where malaria is endemic and thus merit further consideration for field evaluation.

## Supplementary Material

Supplemental material
