# Supplementary material for: Lead Clinical and Preclinical Antimalarial Drugs Can Significantly Reduce Sporozoite Transmission to Vertebrate Populations
Source: Antimicrob Agents Chemother. 2014 Dec 23;59(1):490–7. doi: 10.1128/AAC.03942-14 (PMC4291391; doi:10.1128/AAC.03942-14)
Supplement: Supplemental material [file supp_59_1_490__index.html]

Lead Clinical and Preclinical Antimalarial Drugs Can Significantly Reduce Sporozoite Transmission to Vertebrate Populations — Supplemental material 

# Lead Clinical and Preclinical Antimalarial Drugs Can Significantly Reduce Sporozoite Transmission to Vertebrate Populations

## Supplemental material

**Files in this Data Supplement:**

- Supplemental file 2 -

  Supplemental Figure S2: impact of sulfadiazine on asexual parasitemia and gametocytemia posttreatment.

  PDF, 177K
- Supplemental file 1 -

  Supplemental Dataset S1: raw data from individual experiments assessing the transmission-blocking efficacy of individual drug regimens.

  XLSX, 221K
