# Supplementary material for: Lead Clinical and Preclinical Antimalarial Drugs Can Significantly Reduce Sporozoite Transmission to Vertebrate Populations
Source: Antimicrob Agents Chemother. 2014 Dec 23;59(1):490–7. doi: 10.1128/AAC.03942-14 (PMC4291391; doi:10.1128/AAC.03942-14)
Supplement: Supplemental material [file AAC.03942-14_zac001153623so2.pdf]

Supplementary Information - Figure S2. Impact of Sulfadiazine on asexual parasitemia and gametocytemia post-treatment.

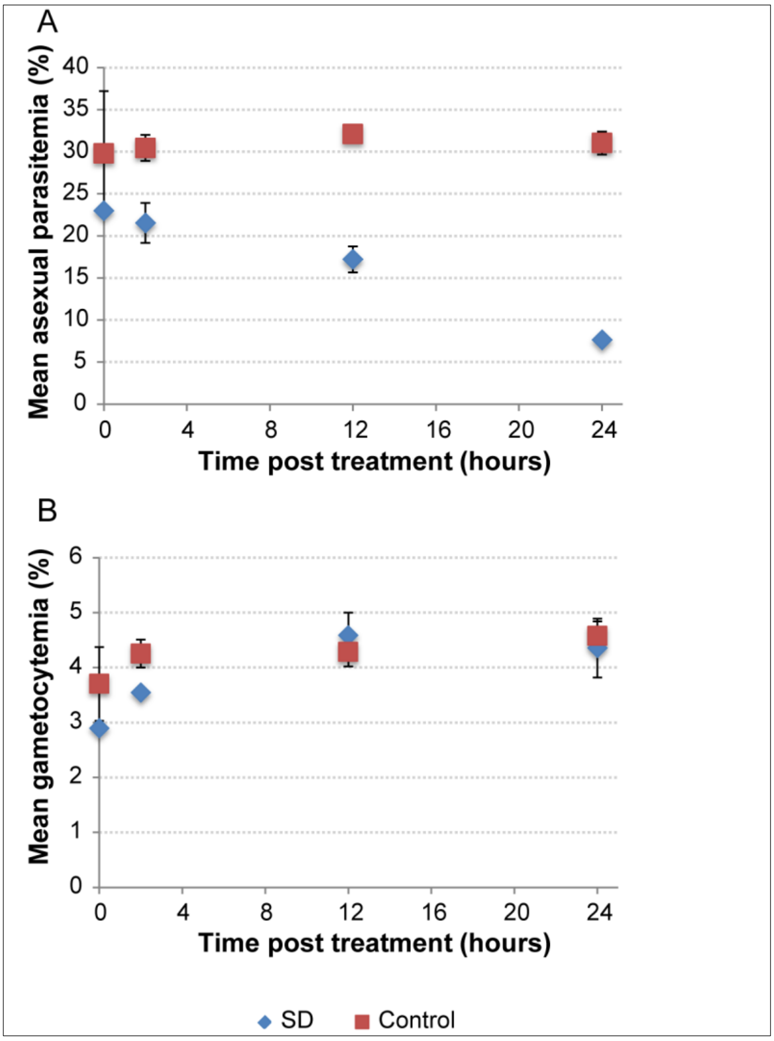

Sulfadiazine treatment was carried out on day 10 *P. berghei* infected mice by *i.p* injection at a dose of 8.4 mg/kg in groups of mice (n=5). Parasitemia and gametocytemia were measured by blood smear at 0, 2, 12 and 24 hours post-treatment. Panel A demonstrates impact on asexual parasitemia whereas panel B shows impact on gametocytemia. Blue points indicate observations made in mice where sulfadiazine treatment was performed, whereas red points indicate control mice where no drug-treatment was performed. Error bars represent the SEM of observations. As demonstrated in panel A, sulfadiazine treatment has a dramatic effect on asexual parasitemia 24 hr post treatment, but no observable effect on gametocytemia (B).
